# Supplementary material for: MetaRibo-Seq measures translation in microbiomes
Source: Nat Commun. 2020 Jun 29;11:3268. doi: 10.1038/s41467-020-17081-z (PMC7324362; doi:10.1038/s41467-020-17081-z)
Supplement: Supplementary file 10 — Supplementary Data 7 [file 41467_2020_17081_MOESM10_ESM.zip › File2/Confidence_High_Taxonomy/57864_out.krona.html]

Javascript must be enabled to view this page.

members
magnitude
magnitudeUnassigned
count
unassigned
taxon
rank

57864\_out

28

superkingdom
2
28

28
phylum
1239

28
186801
class

28
order
186802

family
541000
28

28
genus
1263

species
1637508
1

SRS1041157\_contig\_number\_3335


SRS048870\_contig\_number\_1756
1
2293194
species

1160721
species

SRS014855\_contig\_number\_3495SRS015578\_contig\_number\_21887SRS018133\_contig\_number\_9621SRS019286\_contig\_number\_3780SRS042628\_contig\_number\_10030SRS045826\_contig\_number\_14349SRS104311\_contig\_number\_6449SRS143523\_contig\_number\_16228SRS893259\_contig\_number\_4067SRS893288\_contig\_number\_2928
10

41978
species

SRS011302\_contig\_number\_4833SRS016629\_contig\_number\_1891SRS024663\_contig\_number\_1156SRS050998\_contig\_number\_3077SRS052027\_contig\_number\_2447SRS064276\_contig\_number\_22347SRS076804\_contig\_number\_4421SRS104636\_contig\_number\_15645SRS144362\_contig\_number\_11482SRS147139\_contig\_number\_767SRS893170\_contig\_number\_1694SRS893358\_contig\_number\_9434SRS893378\_contig\_number\_13562
13


SRS014313\_contig\_number\_759SRS018313\_contig\_number\_747SRS077335\_contig\_number\_8091
3
species
2293177
